# Effect of Ultrafiltration of Milk Prior to Fermentation on Mass Balance and Process Efficiency in Greek-Style Yogurt Manufacture

**DOI:** 10.3390/foods7090144

**Published:** 2018-09-04

**Authors:** Adriana Paredes Valencia, Alain Doyen, Scott Benoit, Manuele Margni, Yves Pouliot

**Affiliations:** 1 Department of Food Sciences, STELA Dairy Research Center, Institute of Nutrition and Functional Foods, Laval University, Quebec, QC G1V 0A6, Canada; adriana.paredes-valencia.1@ulaval.ca (A.P.V.); Alain.Doyen@fsaa.ulaval.ca (A.D.); scott.benoit.1@ulaval.ca (S.B.); 2 CIRAIG, Département de Mathématiques et de Génie Industriel, École Polytechnique de Montréal, Montreal, QC H3C 3A7, Canada; manuele.margni@polymtl.ca

**Keywords:** Greek-style yogurt, ultrafiltration, acid whey membrane fouling, energy consumption

## Abstract

Ultrafiltration (UF) can be used to concentrate yogurt to produce Greek-style yogurt (GSY) (UF-YOG), but this generates acid whey permeate, which is an environmental issue. However, when UF is applied before fermentation (UF-MILK), a nonacidified whey permeate is generated. For this study, two model GSYs (UF-YOG and UF-MILK) were produced to compare the composition, UF performance, and energy consumption of the two processes. For UF-MILK, skim milk was ultrafiltered with a 30 kDa spiral-wound UF membrane to achieve a 3× volume reduction factor (VRF). The retentate was fermented to a pH of 4.5. The UF-YOG process was the same except that regular yogurt was ultrafiltered. Both GSYs had similar protein (~10%) and solid content (~17%). As expected, lactic acid/lactate was not detected in UF-MILK permeate, while 7.3 g/kg was recovered from the UF-YOG permeate. Permeation flux values (11.6 to 13.3 L m^−2^ h^−1^) and total flux decline (47% to 50%) were constant during UF-MILK, whereas drastic decreases in these two membrane performance indicators (average flux: 38.5 to 10.9 L m^−2^ h^−1^; total flux decline: 2% to 38%) were calculated for UF-YOG. Moreover, for UF-YOG, UF membrane performance never recovered, even when drastic and repeated cleaning steps were applied. Energy consumption was 1.6 kwh/kg GSY and remained constant for UF-MILK, whereas it increased from 0.6 to 1.5 kwh/kg GSY for UF-YOG. Our results show that, although the composition of GSYs was similar for both processes, the UF step of yogurt concentration affected process efficiency due to drastic and permanent membrane fouling.

## 1. Introduction

Greek-style yogurt (GSY) is currently the largest growing market segment in the dairy industry [1,2]. This commercial success is explained by its rich texture and desirable organoleptic characteristics as well as its nutritional benefits (high-protein and low-fat content). Traditionally, GSY has been produced by straining regular yogurt through a cheesecloth to reach the desired solids level [3]. Until recently, dairy processors mainly used centrifugation or ultrafiltration (UF), a pressure-driven membrane-separation process, to increase the solid content of yogurt [4]. However, these technologies induced the production of large quantities of acid whey, which represents about two-thirds of the initial milk processed [5,6]. Contrary to acid whey recovered after production of acid-coagulated dairy products (fresh cheeses and cream cheese), little specific information is available regarding GSY acid whey composition except that this waste is mainly composed of water (~94%), lactose (~5%), minerals (potassium, phosphorous, and sodium), and lactic acid/lactate generated during the fermentation of milk [1]. Valorization of GSY acid whey is a challenge since it cannot be discharged to surface water due to its high Biological Oxygen Demand (BOD). Moreover, its low protein concentration limits its use in human or animal feed, and high mineral and lactic acid/lactate content had a negative impact on lactose crystallization and quality [7].

Acid-whey management is a growing environmental and economic problem for the dairy industry. Different options were recently proposed to improve GSY acid whey valorization, such as its neutralization and concentration by reverse osmosis for using as a food additive or the use of microbial enzymes to generate galactooligosaccharides [2]. However, the best option would be to produce a GSY without generating acid whey. This can be achieved by protein fortification of conventional yogurt using milk protein or micellar casein concentrates [8,9,10,11]. Another strategy is to produce an ultrafiltered milk retentate that is subsequently fermented to manufacture a protein-enriched yogurt. In this case, the dairy waste generated is a deproteinated sweet whey that can be processed further without issue. 

Several studies have compared the characteristics of the final products from the two UF systems used for the production of protein-enriched yogurt: the fermentation of UF retentate (UF-MILK) and the UF of yogurt (UF-YOG). Tamine et al. [12] demonstrated that labneh (a concentrated yogurt popular in the Middle East) produced by UF-YOG and UF-MILK methods had similar protein and solid contents, but the one obtained using the UF-YOG method had increased firmness. Ozer et al. [13,14] concluded that a shearing effect at the surface of the UF membrane during yogurt concentration had an impact on the elastic and viscous properties of labneh since the process affected gel structure and produced thicker casein strands than in the traditional sample. More recently, Uduwerella et al. [15] have described the use of UF to generate a yogurt base prior to fermentation and straining. It was shown that this approach reduces acid whey discharge to almost nothing when a milk base of 17.5% total solids is used. Efficiency in the manufacture of GSY is defined by several processing characteristics during the concentration of milk and acidification of the media but it is also concerned with the overall mass balance of the milk constituents. The aim of this study was to compare the acidification rate, ultrafiltration performance, energy consumption, and mass balance of milk components for both UF processes (UF-YOG and UF-MILK) used for GSY production.

## 2. Materials and Methods

### 2.1. Greek-Style Yogurt Production

#### 2.1.1. Milk and Starter Culture

**Figure 1:**
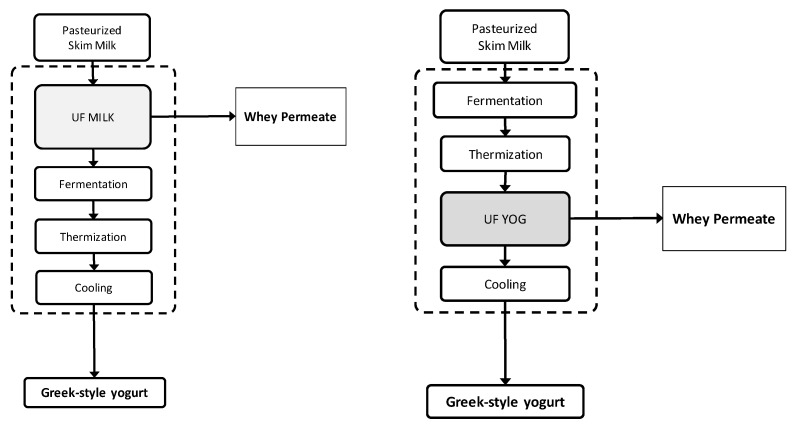
Greek-style yogurt (GSY) processing including ultrafiltration (UF) concentration step.

Commercial pasteurized skim milk (120 kg) purchased from a local dairy supplier (Natrel, QC, Canada) was divided into two equal lots for triplicate (3 × 20 L) productions. The first lot (60 kg) was used for conventional yogurt production (Section 2.2, Figure 1) before the concentration step by UF (UF-YOG). The remaining skim milk (60 kg) was concentrated by UF for skim milk retentate production (UF-MILK) before fermentation. Both samples were inoculated with a freeze-dried mixed strain culture containing *Streptococcus thermophilus* and *Lactobacillus delbrueckii subsp. bulgaricus* (FD-DVS YC-380, 50 U, Chr. Hansen Inc., Fromagex, Rimouski, QC, Canada). 

#### 2.1.2. Ultrafiltration System

A pilot-scale UF system (GEA NIRO, Hudson, WI, USA) with a single membrane cartridge was used for filtration steps. The filtration unit was equipped with a 60 L stainless-steel storage tank, a sanitary positive displacement pump (D/G-10, 576V, 5 HP Wanner International Ltd., Church Crookham, Hampshire, England), two pressure gauges to monitor inlet and outlet pressure and a flow-restriction valve to control transmembrane pressure. The filter was a polyethersulfone (PES) spiral-wound UF membrane (Synder Filtration, Vacaville, CA, USA) with molecular weight cutoff of 30 kDa. For UF-MILK production, a conventional membrane element with 46 mil spacer thickness and 2.04 m^2^ membrane surface area was used. However, for UF-YOG production, a spacer thickness of 80 mil was selected, as recommended by the manufacturer, due to the higher product viscosity. The total membrane surface area was 1.48 m^2^ for that element. All ultrafiltrations were performed at transmembrane pressure (TMP) of 551 kPa (80 psi) and 50 °C. Ultrafiltration membranes were systematically conditioned before each run using the following procedure: a 10 min cleaning step was performed at pH 10.5, 50 °C, with 0.1 mol/L of NaOH (Ultrasil 25™, Ecolab Inc., Laval, QC, Canada) in a closed circuit at zero pressure. If the chlorine concentration was greater than the allowable limit (checked with Iodine-Chlorine test kit #321), the system was rinsed with tap water; otherwise, chlorine was added. This rinsing operation was repeated after 10 min. Pure water flux was measured as an indicator of membrane cleanliness. If low water flux values were observed (>10% difference from clean water flux of that membrane), an additional cleaning cycle was performed to restore membrane permeability. At the end of each filtration experiment, the UF membrane was rinsed. The first cleaning step was performed at pH 10.5 with 0.1 mol/L of NaOH (Ultrasil 25™, Ecolab Inc., Laval, QC, Canada). The second cleaning step was performed with citric acid solution at 0.1% (*v*/*v*) (Ultrasil 76™, Ecolab Inc., Laval, QC, Canada). In some specific cases, enzymatic cleaning (Ultrasil 63™, Ecolab Inc., Laval, QC, Canada) was necessary due to considerable membrane fouling. Between all steps, the system was rinsed with tap water until a neutral pH was reached. The membrane was stored at 4 °C in an acid solution of 0.5% (*v*/*v*) (Ultrasil MP™, Ecolab Inc., Laval, QC, Canada) until use. 

#### 2.1.3. Greek-Style Yogurt Production

Figure 1 depicts the two experimental approaches (UF-MILK and UF-YOG) used to produce GSY at pilot scale.

For UF-YOG, pasteurized skim milk was first heated at 90 °C for 5 min, cooled to 43 °C and then inoculated according to the methodology of Bong and Moraru [10], with some modifications. The starter culture solution was prepared by mixing 3 g of freeze-dried DVS culture with 25 mL of skim milk at 43 °C (12% *w*/*v*). The mixture was incubated at 43 °C for 10 min to acclimate the starter culture. Next, 6 g of the starter culture was mixed with 60 L of skim milk at 43 °C to obtain a final YC-380 concentration of 0.012 g/L, as recommended by the yogurt-culture supplier. The fermentation step was stopped at a theoretical pH of 4.2 and thermization was performed at 60 °C for 80 s to avoid recontamination and to minimize postfermentation. Conventional yogurt was finally cooled at 4 °C before the UF concentration step. After the fermentation step, conventional yogurt (60 L) was heated at 50 °C and transferred into the UF stainless-steel storage tank. A plate heat exchanger was used to maintain the yogurt temperature during the UF step. The yogurt was concentrated in batch mode, i.e., the retentate was recycled back and permeate was continuously removed until a theoretical concentration factor (CF) of 3.0× was reached. Retentate and permeate samples were collected at the end of filtration, and the final volume of retentate was cooled at 4 °C until analysis. For UF-MILK, pasteurized skim milk was ultrafiltered using the same conditions and parameters (TMP, CF) as described for UF-YOG production. At the end of UF, retentate and permeate samples were collected. The remaining retentate was cooled at 43 °C and used for GSY production (UF-MILK) according to the methodology described earlier for fermentation. For both GSY productions (UF-MILK and UF-YOG), a similar decrease rate of 0.33 pH unit/60 min was obtained. The total length of fermentation was 420 min for both processes to reach a final pH ranging from 4.1 to 4.6. 

#### 2.1.4. Permeation Flux and Energy Consumption during UF

Permeation flow rate was measured every 10 min. by weighing permeate collected in a flask during 1 min. until the desired concentration factor. Flux values were calculated as in Equation (1)

J = Fp/S(1)

where J is the permeation flux (kg/h·m^2^); Fp the permeation flow rate (kg/h); and S the membrane surface area (m^2^).

Real-time measurement of voltage and current values were obtained from data recorded by a voltmeter (model Fluke 3000FC, Montréal, QC, Canada) and 3 current clamps (model Fluke A3100FC) directly connected to the induction motor terminals of the UF system’s positive displacement pump. Voltage and current values were automatically recorded every minute during all UF steps. These values were used for real power consumption calculation (Equation (2)) of the 3-phase motor of the pump:

W = √3 × V × I × cos(θ)(2)

where W is the real power (Watt); V is the voltage (Volt); I is the current (Ampere); and cos(θ) is the power factor. The value for cos(θ) was calculated as a function of pump load, according to the manufacturer’s data, and it ranged from 0.54 to 0.55.

Consumption was calculated using Equation (3):

P = (W × t)/1000(3)

where P is the energy consumption (kwh); W is the real power (W); and t is the UF duration (h). 

Energy consumption data were used to compare the energy required to produce a kg of GSY from UF-MILK and UF-YOG experiments.

### 2.2. Rejection Coefficient

The rejection coefficients (σ) of the membrane for protein, lactose, minerals, and lactic acid/lactate were calculated using Equation (4):

σ = 1 − Cp/Cr(4)

where Cp and Cr are the component’s concentration in the permeate and retentate, respectively.

### 2.3. Analytical Methods

The compositions of initial skim milk, UF permeate, and retentate, as well as GSY samples, were obtained by the analytical methods described next. Total solids were measured using the Association of Official Analytical Chemists (AOAC) gravimetry method [16]. Total nitrogen was obtained by combustion using a LECO-FP528 carbon and nitrogen analyzer (LECO, St. Joseph, MI, USA). Nitrogen concentrations in the samples were converted into protein percentages by multiplying the nitrogen result by a conversion factor of 6.38, the value commonly used for milk proteins. Minerals were obtained according to the AOAC method [17]. 

Lactose and acid lactic/lactate concentration were determined through high-performance liquid chromatography (HPLC) (IDF Standard 198/ISO 22662) with a Waters chromatograph (Waters Corp., Milford, MA, USA) equipped with a Hitachi (Foster City, CA, USA) differential refractometer detector, a 600E controller, a column oven, and a cooled 717Plus autosampler. An ICSep ION-300 column (Transgenomic, Omaha, NE, USA) was used with 8.5 mM of H_2_SO_4_ as the mobile phase at a flow rate of 0.4 mL min^−1^. The column temperature was kept constant at 40 °C. External standard calibration was performed for quantifications. 

### 2.4. Statistical Analyses

All experiments were performed in triplicate. The performance of the UF system (rejection coefficient), the compositional differences of initial milks, retentates, permeates, and GSY from UF-MILK and UF-YOG, as well as content of dairy components listed in average mass balance, were subjected to analysis of variance (ANOVA) (*p* < 0.05 as probability level for acceptance) using SAS software version 9.1 (SAS Institute Inc., Cary, NC, USA). 

## 3. Results and Discussion

### 3.1. Rejection Coefficient of Milk Components during UF

Table 1 summarizes the separation performance of the two membrane elements used for UF experiments. Similar rejection coefficients (σ) were observed for the two membranes for protein (0.94 ± 0.01 vs. 0.93 ± 0.01) and lactose (0.16 ± 0.05 vs. 0.17 ± 0.08) during filtration. However, the UF-MILK membrane had a much higher σ (0.69 ± 0.04) for minerals compared to the UF-YOG membrane (σ = 0.18 ± 0.02). This discrepancy can be attributed to the lower pH value of yogurt, which favors a higher proportion of soluble salts [3] and greater permeability of these salts through the UF membrane. The permeability of lactic acid/lactate could not be measured during the UF-MILK process; however, a σ value of 0.18 ± 0.04 was found for UF-YOG, which agrees with the permeability value of 0.17 ± 0.08 found for lactose during the same UF experiments.

### 3.2. Performance Indicators of the Ultrafiltration Process

Table 1 also reports performance indicators calculated during concentration of skim milk (UF-MILK) or yogurt (UF-YOG). The average flux for the first UF replicate (R1) for UF-YOG (38.5 L m^−2^ h^−1^) was about three times higher than UF-MILK (13.3 L m^−2^ h^−1^) after concentration of yogurt and skim milk to a calculated CF of (2.81 ± 0.25)X. Total flux decline and energy consumption were also higher for UF-MILK (47% and 1.6 kwh/kg GSY, respectively) compared to UF-YOG (2% and 0.6 kwh/kg GSY, respectively). Since membrane molecular weight cut-off (MWCO) and material were similar, this difference is explained by the higher spacer thickness of the UF-YOG membrane (80 mil) compared to the one used for UF-MILK (46 mil). The compositional and physicochemical differences between milk and yogurt could also explain why the higher total flux decline is also greater for UF-MILK (47%) than for UF-YOG (2%). For the two other replicates (R2 and R3), all performance indicators (average flux, total flux decline, flux recovery, and energy consumption) calculated during and after concentration of milk by UF membrane (UF-MILK) remained constant. For the UF-YOG membrane, and contrary to UF-MILK, these indicators changed considerably as a function of the experiment. In fact, compared to R1, the average flux decreased by 56% and 72%, while the total flux decline reached 13% and 38% for R2 and R3, respectively. These losses of membrane performance were related to nonrecovery of permeation flux after R2 (<60%) and R3 (<40%), after 5 to 7 cleaning cycles, and caused increased energy consumption during yogurt concentration (0.6 to 1.5 kwh/kg GSY from R1 to R3).

The discrepancies in UF performance between UF-MILK and UF-YOG are difficult to interpret. Very little information is available about UF performance during protein-enriched yogurt production. Tamine et al. [18] determined that the highest permeate flux was obtained at 50 °C. At low filtration temperatures (25 °C to 35 °C), Brazuelo et al. [19] showed that permeate flux improvement was achieved at 25 °C with a high recirculation rate. We show that UF performance is drastically reduced during concentration of yogurt compared to concentration of initial skim milk in terms of the evolution of average flux, total flux decline, and flux recovery upon cleaning, as a function of replicates (R1 to R3). Different hypotheses may also explain these results. The first is that the 80 mil spacer thickness of the UF membrane used for UF-YOG promoted accumulation of precipitated casein grains through the grid structure. This accumulation required prolonged exposure of the membrane to cleaning conditions to completely remove the fouling material [20]. Bouzid et al. [21] showed that, at acidic pH, permeation fluxes during UF at 25 °C (PES material, MWCO of 5–10 kDa) were controlled by casein aggregates after their precipitation in skim milk. Calcium phosphate solubilization at lower pH during milk fermentation could also explain the decrease in membrane performance during yogurt filtration. It has been demonstrated that interactions between membrane material and phosphates, calcium, and organic molecules of whey or milk occur during filtration and induce fouling [21]. More specifically, calcium ions are often involved in protein–protein and protein–membrane interactions [22]. The heating of the milk (90 °C for 5 min) before inoculation could also explain the decrease in membrane performance during yogurt concentration by UF. As extensively reviewed by many authors [23,24,25], thermal denaturation of whey proteins, especially β-lactoglobulin, generates protein aggregates between casein and whey proteins, which dramatically improves yogurt texture in terms of firmness and water-holding capacity. These specific interactions between colloidal and serum proteins at high temperatures are modulated by thiol-disulfide interchange during formation of the beta-lactoglobulin-kappa-casein complex [26]. However, other studies have shown that aggregation of milk proteins are responsible for membrane fouling since the elimination of large aggregates by prefiltration or ultrasonic treatments improved filtration performance [27,28,29]. 

The overall energy consumption for production of 1 kg of GSY, which drastically increased UF-YOG replicates, can be explained by the decrease in membrane performance (average flux, total flux decline) resulting from persistence of membrane fouling after the cleaning step (flux recovery upon cleaning). Obviously, the energy consumption calculated in Table 1 did not account for the extra energy requirements for cleaning. This is especially important since fouling issues were encountered with the UF-YOG membrane element. A different membrane configuration, such as tubular, allowing the UF-concentration of viscous feeds and/or feeds containing suspended particles, would be more appropriate for yogurt concentration [30].

### 3.3. Compositional Characteristics of Initial Skim Milk, Retentates, GSY, and Permeates

Table 2 reports the composition of skim milk, UF retentate and permeate, as well as GSY obtained after skim milk and retentate fermentation by YC-380 culture for both process UF MILK and UF-YOG. The UF-MILK was produced by concentrating skim milk by UF and the retentate was fermented to produce GSY. As expected, total solids, protein, and minerals were increased in the retentate due to concentration by UF. Since lactose rejection was calculated as 0.16 ± 0.05, its concentration also increased in the retentate. The protein concentration in the retentate indicated that the CF reached 2.9×, which is close to the theoretical targeted CF of 3.0×. Protein concentration in the permeate was 0.62 ± 0.05%, representing a rejection coefficient of 0.95, which is expected for a 30 kDa UF membrane. The retentate and GSY had similar compositions, as expected, for lactose and lactic acid/lactate. Indeed, during fermentation the lactose concentration decreased in GSY, while lactic acid/lactate, initially absent in the retentate, increased in the GSY due to the fermentation. 

As presented for UF-MILK, the composition of initial milk and conventional yogurt in the UF-YOG process was similar except for lactose and lactic acid/lactate due to the fermentation step. After concentration of conventional yogurt by UF, protein concentration increased by a factor of 2.6× in GSY, and the rejection coefficient was calculated to be 0.82. Lactic acid/lactate concentration in the permeate was double that in conventional yogurt due to the UF concentration step. 

When UF-MILK and UF-YOG were compared, the water, total solid (TS), and protein contents were not different (*p* > 0.05). However, and compared to UF-MILK, the UF-YOG lactose concentration was higher and minerals and lactic acid/lactate was decreased.

### 3.4. Comparative Mass Balance 

**Figure 2:**
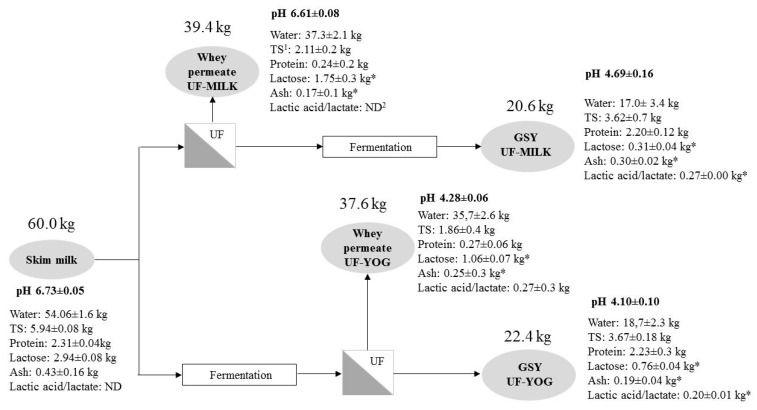
Average mass balance of milk components as influenced by GSY production method. ^1^ Total solid; ^2^ not detected; * significant difference (*p* < 0.05) observed between both permeates or both retentates.

Comparative mass balance is presented in Figure 2. From 60.0 kg of pasteurized skim milk, the two approaches, UF-MILK and UF-YOG, produced 20.6 and 22.4 kg of GSY, respectively. While the final mass of GSY was similar for both processes, some differences in the distribution of milk components in the GSY and coproducts (UF-permeates) must be noted. Concerning byproducts, Permeate from UF-YOG contained a greater proportion of milk salts (0.25 ± 0.30 vs. 0.17 ± 0.10 kg) and lactic acid/lactate (not detected vs. 0.27 ± 0.30 kg) byproducts compared to UF-MILK. It also contained less lactose (1.06 ± 0.07 vs. 1.75 ± 0.30 kg), a difference of 40% between both permeates, and a decrease of 40% (UF-MILK) and 64% (UF-YOG) from the initial lactose content. These decreases mean that the lost lactose will not be available for further processing. For final GSY, and related to the differences observed for byproducts, significant differences were observed for lactose, minerals, and lactic acid/lactate content between UF-MILK and UF-YOG. Indeed, while lactose content was lower (*p* < 0.05) in GSY obtained from UF-MILK (0.31 ± 0.04 vs. 0.76 ± 0.04 kg), mineral (0.30 ± 0.02 vs. 0.19 ± 0.04 kg) and lactic acid/lactate (0.27 ± 0.04 vs. 0.20 ± 0.08 kg) contents were higher (*p* < 0.05) in UF-MILK compared to UF-YOG.

Valorization of lactose contained in acid whey permeate poses a challenge due to the presence of lactic acid/lactate, which interferes with lactose purification [7]. This is important because the most abundant solid from skim milk is lactose and its valorization is key to determining the economic viability of whey permeate processing by ingredients manufacturers [31]. The UF-MILK process allows for generation of a mild permeate likely to facilitate the valorization of its component and, therefore, have potentially higher economic value. 

## 4. Conclusions

The purpose of this study was to evaluate two different technological approaches (UF-MILK and UF-YOG) using UF membranes for GSY production. Our results provided the first evidence that the composition of GSY and coproduct (permeate) and specifically membrane performances were drastically modified as a function of both technological approaches. 

This study suggests that performing UF on milk before fermentation in the manufacture of GSY is beneficial in terms of mass balance because the milk components are better valorized. Since the performance of the 80 mil membrane spacer used for UF-YOG in this study did not produce an optimal UF performance for yogurt, further work should be done to identify a better membrane configuration. 

## Figures and Tables

**Table 1:** Comparative performance of 30 kDa spiral-wound membrane on UF of milk and yogurt.

	**UF-MILK**			**UF-YOG**		
**Rejection Coefficient**						
Protein	0.94 ± 0.01			0.93 ± 0.01		
Lactose	0.16 ± 0.05			0.17 ± 0.08		
Minerals	0.69 ± 0.04 *			0.18 ± 0.02 *		
Lactic acid/lactate	N/A			0.18 ± 0.04		
**Membrane Performance and Fouling**						
	**R1** ^a^	**R2**	**R3**	**R1**	**R2**	**R3**
Average flux (L m^−2^ h^−1^) ^b^	13.3	11.6	12.8	38.5	17.1	10.9
Total flux decline (%) ^c^	47	48	50	2	13	38
**Flux recovery upon cleaning (%) ^d^**	100	100	100	100	<60	<40
Volume concentration factor (CF) ^e^	2.78 ± 0.26					
**Energy consumption for UF (kwh/kg GSY) ^f^**	1.6	1.6	1.7	0.6	1.2	1.5

* *p* < 0.05; ^a^ R = Replicate; ^b^ Average permeation flux arithmetical mean of flux values between CF = 1× and CF = 3×; ^c^ Percentage of permeation flux loss between initial and final values; ^d^ Full cleaning cycles (see materials and methods); ^e^ Target CF value of 3.00; ^f^ Average energy-consumption arithmetical mean of flux values between CF = 1 and CF at the end of the UF concentration step. UF-MILK: UF of skim milk to generate retentate that was fermented to produce GSY; UF-YOG: UF of conventional yogurt to produce GSY.

**Table 2:** Comparative composition of UF fractions and final composition of GSY.

		UF-MILK			UF-YOG		
Component (g/100 g of Milk or UF Fraction)	Milk	Retentate	Permeate	Fermented Retentate (GSY)	Fermented Milk	Retentate (GSY)	Permeate
**Water** ^1^	90.10 ± 0.04 ^a^	82.66 ± 0.3 ^b^	94.65 ± 0.10 ^c^	82.42 ± 0.18 ^b^	90.92 ± 0.11 ^a^	83.60 ± 0.49 ^b^	95.06 ± 0.22 ^c^
**TS**	9.09 ± 0.02 ^a^	17.34 ± 0.38 ^b^	5.35 ± 0.04 ^c^	17.58 ± 0.28 ^b^	9.08 ± 0.05 ^a^	16.4 ± 0.8 ^b^	4.94 ± 0.6 ^c^
**Protein** ^2^	3.85 ± 0.07 ^a^	11.09 ± 0.59 ^b^	0.62 ± 0.05 ^c^	10.68 ± 0.46 ^b^	3.90 ± 0.27 ^a^	9.97 ± 1.46 ^b^	0.71 ± 0.18 ^c^
**Lactose**	4.90 ± 0.02 ^a^	5.24 ± 0.08 ^b^	4.43 ± 0.29 ^a^	1.49 ± 0.10 ^c^	3.50 ± 0.16 ^d^	3.41 ± 0.14 ^d^	2.83 ± 0.28 ^e^
**Minerals**	0.72 ± 0.04 ^a^	1.39 ± 0.15 ^b^	0.42 ± 0.03 ^c^	1.45 ± 0.04 ^b^	0.75 ± 0.01 ^a^	0.83 ± 0.01 ^a^	0.67 ± 0.01 ^a^
**Lactic Acid/Lactate**	ND ^3^	ND	ND	1.29 ± 0.04 ^a^	0.58 ± 0.05 ^b^	0.90 ± 0.05 ^c^	0.73 ± 0.019 ^d^

^1^ Water = 100; TS: total solid; ^2^ protein = total nitrogen × 6.38; ^3^ ND: not detected; different letters indicate significant differences between milk, retentate, and permeate fractions (*p* < 0.05).
